# Supplementary material for: Structure and conformational dynamics of Clostridioides difficile toxin A
Source: Life Sci Alliance. 2022 Mar 15;5(6):e202201383. doi: 10.26508/lsa.202201383 (PMC8924006; doi:10.26508/lsa.202201383)
Supplement: Supplementary file 1 [file LSA-2022-01383_TableS1.docx]

**Table S1 Data collection and refinement statistics.**

| **Data Collection** | **DRBD-5CROPs** |
| --- | --- |
| Space Group | C 1 2 1 |
| Cell Dimensions |  |
| a, b, c (Å) | 379.51, 187.64, 95.32 |
| α, β, γ (°) | 90.00, 101.30, 90.00 |
| Wavelength (Å) | 0.97910 |
| Resolution (Å) | 186.08-3.18 (3.29-3.18) * |
| *R*_merge_ | 0.283 (1.140) |
| *R*_pim_ | 0.177 (0.715) |
| Wilson B-factor (Å^2^) | 59.57 |
| Mean *I/σ(I)* | 4.43 (1.01) |
| *CC_1/2_* | 0.944 (0.380) |
| Completeness (%) | 99.07 (98.65) |
| Redundancy | 3.5 (3.5) |
| **Refinement** |  |
| Resolution (Å) | 186.08-3.18 (3.29-3.18) |
| No. of reflections | 108739 (10768) |
| *R*_work_ ^d^/*R*_free_ | 0.207 (0.310)/0.254 (0.356) |
| No. of atoms | 25621 |
| Protein | 25441 |
| Ligand/ion | 180 |
| Water | 0 |
| B-factor (Å^2^) | 53.0 |
| Protein | 53.2 |
| Ligand/ion | 88.9 |
| Water | 0 |
| Ramachandran plot |  |
| Favored (%) | 92.33 |
| Allowed (%) | 7.67 |
| Outliers (%) | 0.00 |
| R.m.s. deviations |  |
| Bond lengths (Å) | 0.011 |
| Bond angles (°) | 1.220 |

* Values in parentheses are for the highest-resolution shell.
