# Supplementary material for: Structure and conformational dynamics of Clostridioides difficile toxin A
Source: Life Sci Alliance. 2022 Mar 15;5(6):e202201383. doi: 10.26508/lsa.202201383 (PMC8924006; doi:10.26508/lsa.202201383)
Supplement: Supplementary file 2 [file LSA-2022-01383_TableS2.docx]

**Table S2 Inter-domain interactions between the β-hairpin of SGS and the α1/2 of pore-forming region.**

| **β-hairpin** | **α1/2** | **Type of Interaction** |
| --- | --- | --- |
| T940 | K1065 | HB (sc-sc), vdW |
| D941 | E1061 | vdW |
|  | K1065 | HB (mc-sc, sc-sc), SB, vdW |
| V942 | A1046 | vdW |
|  | L1058 |  |
|  | E1061 |  |
|  | L1062 |  |
|  | K1065 |  |
| N943 | D1055 | vdW  vdW  vdW  HB (mc-sc), vdW |
|  | L1057 |  |
|  | L1058 |  |
|  | E1061 |  |
| N945 | E1049 | HB (sc-sc), vdW |
| L947 | I1041 | vdW |
|  | N1042 |  |
|  | L1043 |  |
|  | A1046 |  |
|  | V1066 |  |
| D948 | G1040 | vdW  vdW  HB (mc-mc, sc-sc), vdW |
|  | I1041 |  |
|  | N1042 |  |
| N949 | G1040 | vdW |
| I950 | G1040 | HB (mc-mc), vdW  vdW |
|  | N1042 |  |

“SB”, “vdW”, and “HB” stand for salt bridge, van der Waals interaction, and hydrogen bond, respectively. “mc” indicates the main-chain-mediated contacts, and all the other contacts are mediated by side-chain atoms.
