# Supplementary material for: Structure and conformational dynamics of Clostridioides difficile toxin A
Source: Life Sci Alliance. 2022 Mar 15;5(6):e202201383. doi: 10.26508/lsa.202201383 (PMC8924006; doi:10.26508/lsa.202201383)
Supplement: Supplementary file 3 [file LSA-2022-01383_TableS3.docx]

**Table S3 Inter-domain interactions at interface I between the CROPs and the DRBD.**

| **hinge** | **DRBD** | **Type of interaction** | **CROPs** | **DRBD** | **Type of interaction** |
| --- | --- | --- | --- | --- | --- |
| E1825 | H954 | vdW | G1832 | G1574 | vdW |
|  | D1624 | vdW | L1833 | G1574 | HB (mc-mc) |
|  | R1651 | HB (sc-sc), SB, vdW |  | H1575 | vdW |
| D1826 | H954 | HB (sc-sc), SB, vdW |  | N1577 | vdW |
|  | S1647 | vdW |  | T1578 | HB (mc-mc) |
|  | G1650 | vdW | I1834 | N1577 | vdW |
|  | R1651 | HB (sc-sc), SB, vdW |  | N1628 |  |
|  | N1686 | vdW | N1835 | T1578 | HB (sc-sc) |
| K1828 | G1650 | vdW | Y1842 | N1626 | vdW |
|  | R1651 |  |  | K1627 | vdW |
|  | Y1684 |  |  | N1628 | HB (mc-sc) |
|  | I1685 |  |  | D1573 | HB (sc-mc) |
|  | N1686 |  | P1845 | D1573 | vdW |
| V1830 | N1577 | vdW |  | E1709 | vdW |
| K1831 | H1576 | vdW |  | Y1710 | HB (mc-sc) |
|  | N1577 | HB (mc-sc) | F1848 | Y1710 | vdW |
|  | R1683 | vdW | T1856 | D1573 | vdW |
|  | Y1710 | vdW | I1857 | S1572 | vdW |
|  | | |  | D1573 | vdW |
|  |  |  |  | H1575 | vdW |
|  |  |  | I1858 | N1571 | HB (sc-mc) |
|  |  |  |  | S1572 | vdW |
|  |  |  |  | D1573 | HB (mc-sc) |
|  |  |  | Y1862 | H1575 | vdW |
|  |  |  | D1888 | N1580 | vdW |

“SB”, “vdW”, and “HB” stand for salt bridge, van der Waals interaction, and hydrogen bond, respectively. “mc” indicates the main-chain-mediated contacts, and all the other contacts are mediated by side-chain atoms.
