# Supplementary material for: Structure and conformational dynamics of Clostridioides difficile toxin A
Source: Life Sci Alliance. 2022 Mar 15;5(6):e202201383. doi: 10.26508/lsa.202201383 (PMC8924006; doi:10.26508/lsa.202201383)
Supplement: Supplementary file 4 [file LSA-2022-01383_TableS4.docx]

**Table S4 Inter-domain interactions at interface II between CROPs and the DRBD.**

| **CROPs** | **DRBD** | **Type of interaction** |
| --- | --- | --- |
| H2234 | K1118 | vdW |
| Y2244 | V1239 | vdW |
|  | P1240 |  |
| S2246 | P1240 | vdW |
|  | G1241 |  |
| Y2247 | T1120 | vdW  vdW  vdW  HB (sc-mc)  vdW |
|  | G1241 |  |
|  | L1242 |  |
|  | S1244 |  |
|  | L1245 |  |
| D2248 | S1244 | vdW |
| Q2252 | P1240 | vdW |
|  | G1241 |  |
| Y2255 | A1238 | vdW |
|  | F1272 |  |
| I2256 | A1238 | vdW |
|  | P1240 |  |
| T2257 | G1237 | vdW |
|  | A1238 | HB (sc-mc, mc-mc) |
| E2259 | D1117 | vdW |
| R2260 | D1117 | vdW |
|  | E1235 | vdW |
|  | T1278 | HB (sc-sc) |
| P2281 | F1272 | vdW |
|  | Y1274 |  |

“vdW” and “HB” stand for van der Waals interaction and hydrogen bond, respectively. “mc” indicates the main-chain-mediated contacts, and all the other contacts are mediated by side-chain atoms.
