# Supplementary material for: Structure and conformational dynamics of Clostridioides difficile toxin A
Source: Life Sci Alliance. 2022 Mar 15;5(6):e202201383. doi: 10.26508/lsa.202201383 (PMC8924006; doi:10.26508/lsa.202201383)
Supplement: Supplementary file 5 [file LSA-2022-01383_TableS5.docx]

**Table S5 Parameters were extracted from SC (Fig. 6) and EC (Fig. S5) FRET histograms fitted with three Gaussian distribution fitting.**

| Variants | pH | Low FRET | | Medium FRET | | High FRET | |
| --- | --- | --- | --- | --- | --- | --- | --- |
|  |  | Mean ±Width | Population (%) | Mean ±Width | Population (%) | Mean ±Width | Population (%) |
| SC | 7 | 0.00 ± 0.09 | 20 | 0.24 ± 0.23 | 20 | 0.62 ± 0.21 | 60 |
|  | 5 | 0.04 ± 0.11 | 40 | 0.23 ± 0.21 | 40 | 0.59 ± 0.37 | 20 |
| EC | 7 | 0.00 ± 0.10 | 60 | 0.17 ± 0.17 | 30 | 0.56 ± 0.51 | 10 |
|  | 5 | 0.01 ± 0.09 | 68 | 0.13 ± 0.13 | 24 | 0.49 ± 0.41 | 8 |
