# Supplementary material for: Structure and conformational dynamics of Clostridioides difficile toxin A
Source: Life Sci Alliance. 2022 Mar 15;5(6):e202201383. doi: 10.26508/lsa.202201383 (PMC8924006; doi:10.26508/lsa.202201383)
Supplement: Supplementary file 6 [file LSA-2022-01383_TableS6.docx]

**Table S6 State mean dwell time values extracted from the three-state fitting model by ebFRET software package** (van de Meent et al 2014)**.**

| Variants | pH | Dwell time (s) | | | | | |
| --- | --- | --- | --- | --- | --- | --- | --- |
|  |  | Low FRET | | Medium FRET | | High FRET | |
|  |  | Mean | 1 σ Range | Mean | 1 σ Range | Mean | 1 σ Range |
| SC | 7 | 0.5 | (0.2, 1.3) | 0.8 | (0.4, 1.6) | 2.9 | (1.8, 4.6) |
|  | 5 | 0.9 | (0.4, 2.3) | 0.4 | (0.2, 1.0) | 0.5 | (0.2, 1.3) |
| EC | 7 | 0.4 | (0.2, 1.0) | 0.4 | (0.2, 0.8) | 2.6 | (1.6, 4.1) |
|  | 5 | 0.9 | (0.4, 2.3) | 0.6 | (0.3, 1.2) | 1.3 | (0.7, 2.6) |
